# Angelicae Dahuricae Radix Inhibits Dust Mite Extract-Induced Atopic Dermatitis-Like Skin Lesions in NC/Nga Mice

**DOI:** 10.1155/2012/743075

**Published:** 2012-02-12

**Authors:** Hoyoung Lee, Jun Kyoung Lee, Hyekyung Ha, Mee-Young Lee, Chang-Seob Seo, Hyeun Kyoo Shin

**Affiliations:** Herbal Medicine EBM Research Center, Korea Institute of Oriental Medicine, 483 Expo-ro, Daejeon Yuseong-gu, 305-811, Republic of Korea

## Abstract

We examined whether Angelicae Dahuricae Radix (AR) suppresses the development of atopic dermatitis (AD)-like skin lesions induced by *Dermatophagoides farinae* in NC/Nga mice. To investigate the effect of AR, we measured the AD severity score, measured plasma levels of IgE and histamine, and performed histological analysis in NC/Nga mice. We also confirmed the anti-inflammatory effects of AR by measuring TARC/CCL17 production from LPS-treated RAW 264.7 cells and mRNA levels of TARC and MDC/CCL22 in TNF-**α**/IFN-**γ**-treated HaCaT cells. 10 mg/day of AR extract was applied for 4 weeks to NC/Nga mice. Both the AR extract and 0.1% tacrolimus suppressed the development of AD-like skin lesions and reduced dermatitis scores of the back and ear skin. AR extracts caused an inhibition of histological changes induced by repeated application of *D. farinae* and a reduction of IgE and histamine levels in plasma (*P* < 0.05). Furthermore, NO production in LPS-treated RAW 264.7 cells was diminished in a dose-dependent manner, and hTARC production and TARC and MDC mRNA levels in TNF-**α**/IFN-**γ**-treated HaCaT cells were diminished by AR. The inhibitory effect of AR on NO, TARC and MDC production may be associated with the suppression of AD-like skin lesions in *D. farinae*-induced NC/Nga mice.

## 1. Introduction

Angelicae Dahuricae Radix (AR) is the dried root of *Angelica dahurica* (F_ISCH._) B_ENTH._ et H_OOKER_ F (Umbelliferae). AR has been used for the treatment of colds, headaches, toothaches, coryza, and psoriasis in Korean traditional medicine [1]. There have been reports that AR has anti-inflammatory [2], antioxidant [3], and cytochrome P450 activity [4]. The major compounds of AR are imperatorin and coumarin. These compounds have also been shown to be effective agents for anticancer responses [5] as well as for the induction of beta-endorphin [6], antihistamine [7], and antioxidative activity [8].

Atopic dermatitis (AD) is a chronic, relapsing skin disorder involving the interplay between migrating lymphocytes and epidermal keratinocytes [9]. AD is a clinical syndrome that is characterized by pruritic and dry skin lesions. Although topical steroids and immunosuppressive drug therapies are used to treat AD patients, these therapies result in adverse effects. At present, many researchers are exploring drugs traditionally used in herbal medicine, which have few adverse effects and excellent efficacy for dermatology [10]. Our study shows that AR is beneficial for treating AD-like skin lesions induced by the dust mite *Dermatophagoides farinae* on NC/Nga mice.

Th2 cells are induced to migrate to specific anatomic locations by thymus- and activation-regulated chemokines (TARC/CCL17) and macrophage-derived chemokines (MDC/CCL22), and regulated on activation which are specific ligands for CCR4 [11, 12]. TARC and MDC have been found to be associated with AD in many reports. The serum levels of TARC and MDC have been shown to be markedly elevated and to correlate with disease severity in patients with AD [13, 14]. In the present study, we examined the effect of AR on *D. farinae*-induced AD-like skin lesions on NC/Nga mice. We also confirmed the anti-inflammatory effect of AR on LPS-induced RAW 264.7 cells and the antiallergy effect of AR on recombinant human tumor necrosis factor-*α* (TNF-*α*)/recombinant human interferon- *γ* (IFN-*γ*)-treated HaCaT cells. 

## 2. Materials and Methods

### 2.1. Animals

Eight-week-old male NC/Nga mice were purchased from Central Laboratory Animal Inc. (Seoul, Korea) and housed in an air-conditioned room maintained at 24 ± 2°C and 55 ± 15% humidity. All procedures involving animals were conducted in accordance with the guidelines of the Institutional Animal Care and Use committee of the Korea Institute of Oriental Medicine (no. 09-173).

### 2.2. Chemicals and Reagents

Oxypeucedanin, oxypeucedanin hydrate, and byakangelicin were isolated from *A. dahurica* by a series of chromatography procedures. The purity of each compound was determined to be above 95% by HPLC analysis. Nodakenin was obtained from Wako (Osaka, Japan). Imperatorin and isoimperatorin were purchased from ChromaDex (Santa Ana, CA, USA). HPLC-grade reagents, methanol, acetonitrile, and water were obtained from J.T. Baker (Phillipsburg, NJ, USA). Other chemicals were of analytical grade. A *D. farinae* ointment was purchased from Biostir Inc. (Hyogo, Japan). The positive control animals were treated with a 0.1% tacrolimus, an immunosuppressive drug (Protopic, Astellas, NY, USA). Human recombinant TNF-*α*, IFN-*γ*, and TARC enzyme-linked immunosorbent assay (ELISA) kits were obtained from R&D Systems (MN, USA). Forskolin, N*^G^*-methyl-L-arginine (NMMA) and silymarin were purchased from Sigma-Aldrich (MO, USA). Mouse IgE and histamine ELISA kits are the products of Bethyl Laboratories Inc. (TX, USA) and Oxford biomedical research (MI, USA), respectively.

### 2.3. Preparation of AR Extract and Analysis of Chemical Components

Two-hundred grams of Angelicae Dahuricae Radix (AR; H-MAX, Korea) was obtained and extracted using a 10-fold volume (w/v) of extraction solvent (70% ethanol) to prepare the liquid extract. The extract was concentrated and then freeze dried to prepare a powdered extract (62.19 g). The extraction yield was 31.10%.

HPLC was used to analyze the chemical components of the AR extract. The chemicals used to identify compounds in the AR extract included the following: oxypeucedanin, oxypeucedanin hydrate, byakangelicin, nodakenin, imperatorin, and isoimperatorin. The HPLC retention times of the six main coumarin compounds were approximately 8.9, 14.4, 14.9, 23.3, 28.3, and 31.1 min for nodakenin, oxypeucedanin hydrate, byakangelicin, oxypeucedanin, imperatorin, and isoimperatorin, respectively [15]. 

### 2.4. Sensitization

AD-like skin lesions were induced in 10-week-old male NC/Nga mice using *D. farinae* extract as described by the manufacturer [16]. An ointment of *D. farinae* extract (Biostir AD) was purchased from Biostir Inc. (Hyogo, Japan) and applied twice per week for 4 weeks. Briefly, the hair on the upper back was shaved, and then 200 *μ*L of 10% (w/v) sodium dodecyl sulfate (SDS) was applied for barrier disruption to the shaved dorsal skin and both surfaces of each ear until the second treatment. After the second treatment, 200 *μ*L of 4% (w/v) SDS was applied to the skin and ears before topical application of 50 mg of Biostir AD. At the start of the experiment, mice were randomized to one of four groups (*n* = 5 each): untreated controls (70% ethanol; Control), the Biostir AD-treated group (50 mg/mouse; *D. farinae*), the Biostir AD with Protopic-treated group (50 mg/mouse; tacrolimus), and the Biostir AD with AR extract-treated group (10 mg/mouse/70% ethanol; AR). AR extract was dissolved into 70% ethanol. Ethanol (70%) was given to the animals in the vehicle control group.

### 2.5. Dermatitis Score

The severity of dermatitis was investigated macroscopically. The back skin and skin lesions on both ears were scored according to the following symptoms: erythema/hemorrhage, edema, excoriation/erosion, and scaling/dryness. The severity score was defined as the sum of individual scores, graded as follows: 0 (no symptom), 1 (mild), 2 (moderate), and 3 (severe).

### 2.6. Histopathology

After sacrificing the mice, the back skin and both ears were fixed in 10%  (v/v) neutral buffered formalin for 24 hr at 4°C. Tissues were next embedded in paraffin and thin sectioned (4 *μ*m thickness). Sections were then stained with H&E solution (hematoxylin and eosin, Sigma-Aldrich Inc., MO, USA) and subsequently mounted under cover slips using Dako-mounting medium (Dakocytomation, Denmark). Photographs were captured using a photometric Quanix digital camera, and images were assembled in Adobe Photoshop. To measure mast cell infiltration, skin sections were stained with toluidine blue, and the numbers of mast cells in four randomly chosen regions were counted.

### 2.7. ELISA

Blood samples were obtained from mice, and plasma was separated by centrifugation at 10,000 rpm for 10 min at 4°C. Plasma was collected and stored at −80°C. Total plasma IgE and histamine levels from treated NC/Nga mice were measured by ELISA according to the manufacturer's instructions.

### 2.8. Cell Culture

The murine macrophage cell line RAW 264.7 was obtained from the American Type Culture Collection (ATCC, Rockville, MD, USA). The cells were maintained in DMEM media containing 5.5% FBS and 1% P&S (100 U/mL of penicillin and 100 *μ*g/mL of streptomycin) in a humidified 5% CO_2_ atmosphere. The human keratinocyte cell line HaCaT, provided by professor Nakyung Lee at Sejeong University, was cultured in DMEM with 10% (v/v) FBS and 1% P&S. RAW 264.7 cells and HaCaT cells were seeded at a density of 2.5 × 10^3^ cells/well and 1.0 × 10^3^ cells/well, respectively, for proliferation analysis for 24 hr. The cell counting Kit-8 reagent (CCK-8, Dojindo, Japan) was added and incubated for 4 hr. We measured the absorbance at 450 nm using a Benchmark Plus ELISA reader (BIO-RAD, Japan), and the percentages of proliferating cells were calculated.

### 2.9. Measurement of Nitric Oxide (NO)

RAW 264.7 cells were seeded at a density of 2.5 × 10^5^ cells in a 48-well plate for the NO assay. After 18 hour incubation, the cells were stimulated with 1 *μ*g/mL of LPS in the presence or absence of AR extracts for the indicated periods. A griess reagent system (Promega. WI. USA) was used to measure the production and inhibition of NO in culture supernatants. Briefly, a 50-*μ*L/well sample was incubated at room temperature with 1% sulfanilamide for 10 min and 1%  *α*-naphthylamine for 10 min. The absorbance was evaluated at 535 nm using a calibration curve with standard.

### 2.10. Measurement of hTARC

HaCaT cells were seeded at a density of 1.0 × 10^6^ cells/well in 6-well plates for the hTARC assay. After 24 hr, the cells were washed and incubated with 1 mL serum-free media containing TNF-*α* (10 ng/mL)/IFN-*γ* (10 ng/mL) plus AR extract for 24 hr. Cells were subsequently incubated with AR extract (200, 500, or 1000 *μ*g/mL) for 24 hr; other groups of cells were treated with forskolin (10 and 30 *μ*M) and silymarin (20 and 50 *μ*g/mL) as positive controls. The absorbance was evaluated using a Benchmark Plus ELISA reader at 450 nm using a calibration curve with a standard.

### 2.11. Gene Expression (RT-PCR)

After a 24-hr incubation of cells as described above, total RNA was isolated using TRIzol reagent (Invitrogen Life Technologies, CA, USA). cDNA was synthesized from total RNA (2 *μg*) using an iScript Select cDNA Synthesis kit (Bio-Rad, CA, USA) according to the manufacturer's instructions. For PCR amplification, we used the following specific primers for the mRNAs: TARC (forward: 5′-actgctccagggatgccatcgttttt-3′, reverse: 5′-acaaggggatgggatctccctcactg-3′); MDC (forward: 5′-aggacagagcatggctcgcctacaga-3′, reverse: 5′-taatggcagggaggtagggctcctga-3′); hGAPDH (forward: 5′-aagggtcatcatctctgccc-3′, reverse: 5′-gtgatggcatggactgtggt-3′), which were expected to yield fragments of 270 bp, 362 bp, and 204 bp, respectively.

### 2.12. Statistical Analysis

Data are reported as the means  ±  SEM and were compared by ANOVA followed by the Bonferroni multiple comparison method. For score data analysis, data were compared by Wilcoxon statistics after Kruskalis-Wallis test (SYSTAT 10.0., SPSS Inc., USA) A *P*-value < 0.05 was defined as statistically significant.

## 3. Results

### 3.1. Histological Analysis

**Figure 1:**
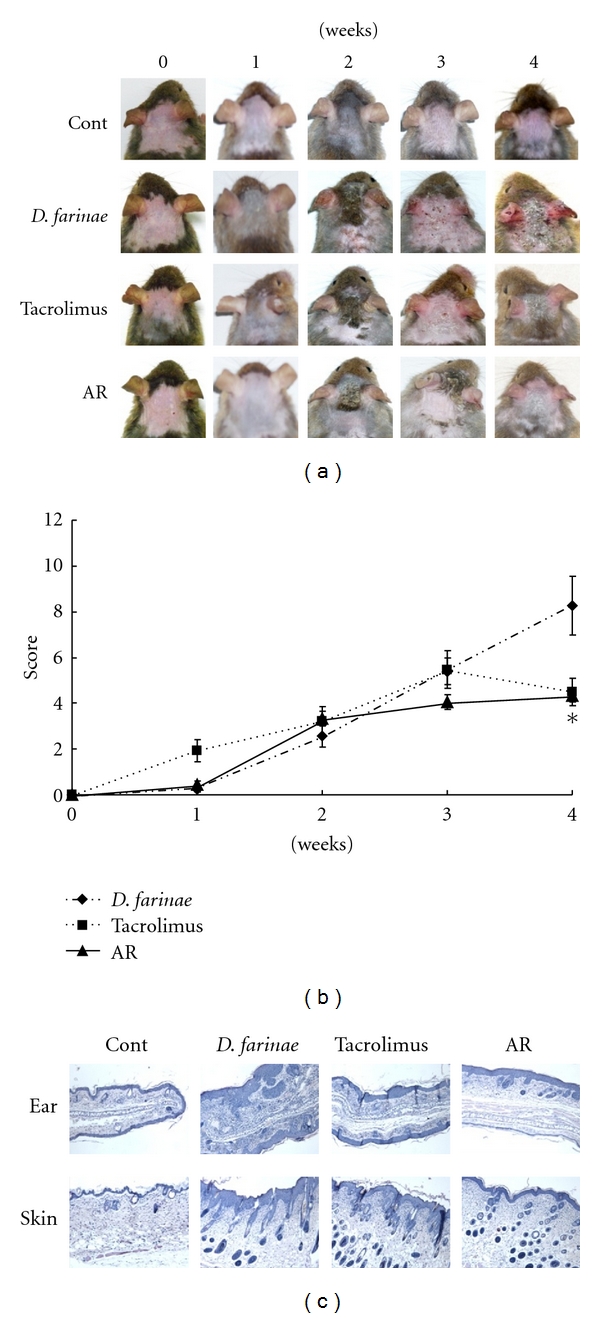
Severity scores and histological changes of treated backs and ears in NC/Nga mice. (a) Representative features and histological changes following consecutive administration of Angelicae Dahuricae Radix (AR) or tacrolimus to *Dermatophagoides farinae*-induced AD-like lesions on the back and ears. The images show the back and ears 4 weeks after sensitization. (b) Effect of consecutive administration of AR or tacrolimus on dermatitis score of *D. farinae*-induced AD-like lesions on the back and ears. AR or tacrolimus were administrated once daily for 4 weeks. (c) Histological features of AD-like lesions treated with AR. The dermatitis scores were evaluated by the procedures described in Materials and Methods (mean ± SEM (*n* = 5), *: *P* < 0.05, compared with* D. farinae*-induced group).

Skin from treated NC/Nga mice was investigated once a week for 4 weeks (Figure 1(a)). The control group showed no physical signs of dermatitis, erythema/hemorrhage, edema, excoriation/erosion, or scaling/dryness. In contrast, the severity scores of the *D. farinae* group gradually increased after sensitization. The groups receiving tacrolimus and AR treatments both showed a reduction in the development of AD-like skin lesions, and a significant difference in lesions was observed between 2 and 4 weeks. The severity scores are shown in Figure 1(b). We show that the tacrolimus and AR treatments suppressed AD-like skin lesions compared with treatments with *D. farinae* alone.

### 3.2. Histopathological Observation

Histopathological findings from the back skin and ear skin are shown in Figure 1(c). Topical application of *D. farinae* increased the thickness of back and ear skin. In the tacrolimus- and AR-treated groups, there were fewer prominent inflammatory changes, such as erosion and hyperplasia of epidermal and dermal tissues.

### 3.3. Plasma Levels of Histamine and IgE

**Figure 2:**
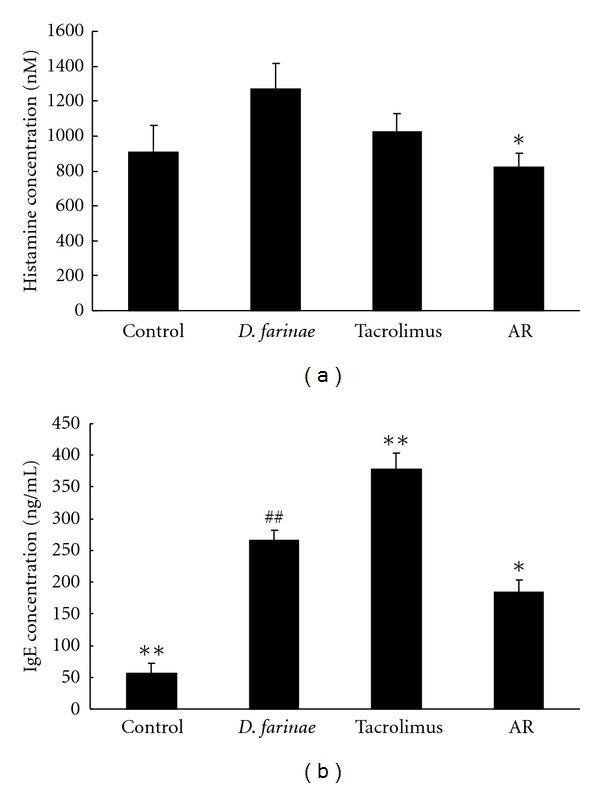
Effect of Angelicae Dahuricae Radix (AR) on plasma levels of histamine and IgE in *Dermatophagoides farinae*-induced AD-like lesions in NC/Nga mice. The groups were untreated (cont), *D. farinae* plus vehicle-treated (*D. farinae*), *D. farinae* plus tacrolimus-treated (tacrolimus), and *D. farinae* plus AR-treated (AR) NC/Nga mice. The plasma concentrations of histamine (a) and IgE (b) were measured by ELISA (mean ± SEM, *n* = 5), ^#^: *P* < 0.05, ^##^: *P* < 0.01 compared with control group, *: *P* < 0.05, **: *P* < 0.01 compared with* D. farinae*-induced group.

The histamine level in plasma is shown in Figure 2(a). The *D. farinae*-treated group (1.27 ± 0.15 *μ*M) had an increase in histamine levels compared with the control group (0.91 ± 0.16 *μ*M) (*P* < 0.01). AR treatment reduced the histamine level significantly (0.82 ± 0.0.08 *μ*M; *P* < 0.05). The group receiving tacrolimus also had decreased histamine levels (1.03 ± 0.10 *μ*M), but this decrease did not reach statistical significance.

Similar trends were seen with IgE levels in plasma. The control group secreted 56.67 ± 14.91 ng/mL IgE. However, the *D*. *farinae*-treated group had a 4.7-fold increase in IgE plasma level (266.54 ± 14.92 ng/mL). When AR was applied to the AD-like lesions, the IgE level was reduced 1.44-fold (184.46 ± 19.70 ng/mL). However, there was no significant difference in IgE levels in the tacrolimus group compared with *D*. *farinae*-treated group (Figure 2(b)).

### 3.4. Effects of AR on NO and hTARC Production

**Figure 3:**
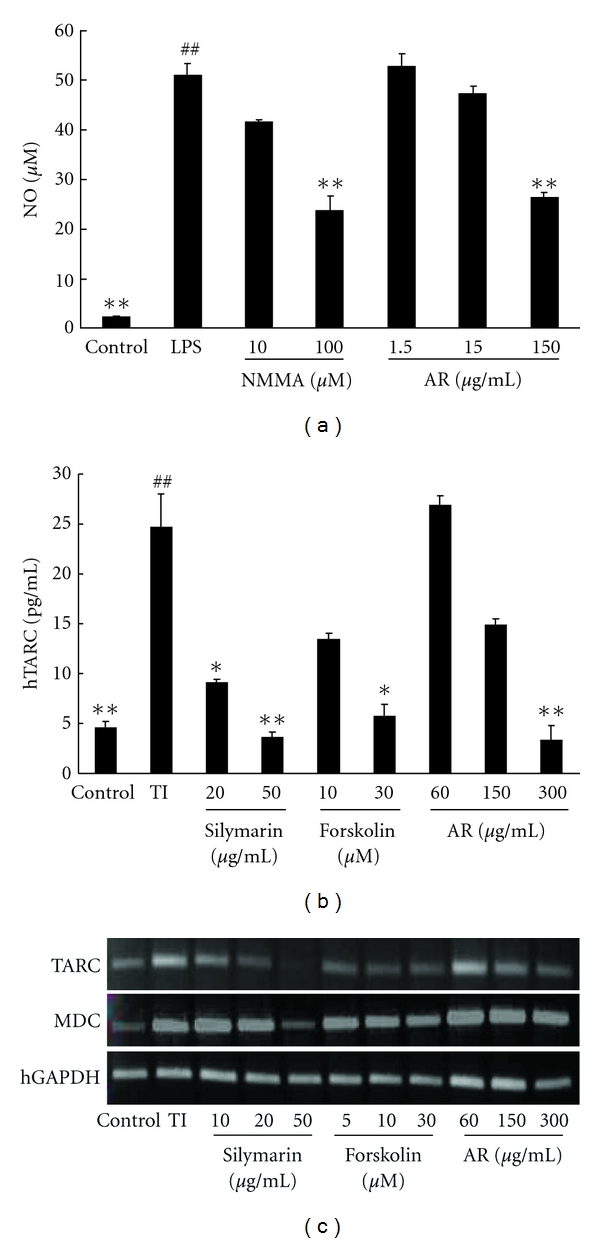
Inhibitory effects on NO and TARC by Angelicae Dahuricae Radix (AR). The concentrations of NO and TARC in the supernatant were determined using NO and TARC ELISA assays. (a) RAW 264.7 cells were treated with LPS (1 *μ*g/mL) for 18 hr. AR inhibited NO production in a concentration-dependent manner (mean ± SEM (*n* = 5), ^#^: *P* < 0.05, ^##^: *P* < 0.01 compared with control group, *: *P* < 0.05, **: *P* < 0.01 compared with the LPS-treated group). (b) HaCaT cells were treated with TNF-*α* plus IFN-*γ* (TI) (10 ng/mL each) for 24 h. TARC levels were significantly suppressed by AR (mean ± SEM, *n* = 5), ^#^: *P* < 0.05, ^##^: *P* < 0.01 compared with control group, *: *P* < 0.05, **: *P* < 0.01 compared with TI treatment. (c) The MDC and TARC mRNA expression levels were determined by RT-PCR analysis, and AR suppressed the mRNA expression of MDC and TARC. The cells were treated with silymarin (10, 20, or 50 *μ*g/mL), forskolin (5, 10, or 30 *μ*g/mL), or AR (60, 150, or 300 *μ*g/mL).

The effects of AR on NO production were examined in LPS-induced RAW 264.7 cells. Control cells released 2.21 ± 0.18 *μ*M of nitrite during 18 hr of incubation. Upon exposure to LPS alone, the cells released 51.01 ± 2.28 *μ*M nitrite (Figure 3(a)). For the positive control, NMMA inhibited LPS-induced nitrite production in a dose-dependent manner, corresponding to a 20% inhibition at 10 *μ*M and a 56% inhibition at 100 *μ*M. Additionally, NO production was inhibited by AR in a dose-dependent manner, corresponding to an 8% inhibition at 15 *μ*g/mL and a 51% inhibition at 150 *μ*g/mL.

The effects of AR on hTARC production were examined in TNF-*α*/IFN-*γ*-induced HaCaT cells. As a positive control for decreasing hTARC levels, we used silymarin (20, 50 *μ*g/mL) and forskolin (10, 30 *μ*M) to inhibit cAMP. The control cells secreted 4.64 ± 0.54 pg/mL hTARC. When each cell type was treated with 10 ng/mL of TNF-*α*/IFN-*γ*, the hTARC level increased 5.3-fold (24.72 ± 3.24 pg/mL). We confirmed that the positive controls induced decreased levels of hTARC in a dose-dependent manner; silymarin-treated cells secreted 9.14 ± 0.29 pg/mL at 20 *μ*g/mL and 3.63 ± 0.56 pg/mL at 50 *μ*g/mL, and forskolin-treated cells secreted 13.50 ± 0.54 pg/mL at 10 *μ*M and 5.72 ± 1.23 pg/mL at 30 *μ*M. AR inhibited TNF-*α*/IFN-*γ*–induced hTARC production in a dose-dependent manner, corresponding to 26.89 ± 1.00 pg/mL, 14.95 ± 0.56 pg/mL, and 3.41 ± 1.40 pg/mL at 60, 150 and 300 *μ*g/mL, respectively (Figure 3(b)).

### 3.5. AR Inhibits the Expression of TARC, MDC, and RANTES

We measured the ability of AR to inhibit transcription of TARC and MDC using RT-PCR. Expression of TARC and MDC was stimulated with TNF-*α*/IFN-*γ* treatment of HaCaT. In the positive controls, silymarin and forskolin suppressed the expression of TARC and MDC. AR inhibited the expression of TARC and MDC in a dose-dependent manner (Figure 3(c)).

## 4. Discussion

In this study, we investigated whether AR suppressed the induction of AD-like skin lesions in *D. farinae*-induced NC/Nga mice. The NC/Nga mouse model is one of the most valuable mouse models of human AD [17]. This model is used to evaluate drug candidates to treat AD. AD is an inflammatory disorder that is caused by genetics factors and environmental factors such as dust mites and mold.

Our study confirms that AD-like lesions such as erythema/hemorrhage, edema, excoriation/erosion, and scaling/dryness are observed in the *D. farinae*-induced model. In this model, AR significantly inhibited the development of AD-like skin lesions on both the back and ears of mice. The histological features of the AR-treated group were similar to those of the control group. These results demonstrate that topical AR application has an anti-inflammatory effect on the AD-like skin lesions on NC/Nga mice. AR also suppressed the histamine and IgE levels in plasma to levels comparable to those of the control group. We confirmed that the AR 10 mg/mouse suppressed the AD-like skin lesions, so further experiments carry out application the dose-dependent of AR extracts and fractions.

Immunosuppressive drugs are very effective in the treatment of AD. Two such drugs, tacrolimus and cyclosporine (CsA), bind calcineurin and inhibit nuclear factor of activated T-cell (NF-AT) nuclear translation [18]. Tacrolimus (0.1%, w/v) is used to treat patients with serious AD over a short period of time, and it suppresses pruritus in AD-like lesions. This treatment also avoids direct application onto inflammation lesions. However, we found that because of adverse effects, this compound is not suitable for use on injuries. In our study, tacrolimus inhibited the increased histamine levels in plasma related with pruritus, whereas the IgE levels were increased by tacrolimus. Hanifin et al. reported that the most common application site adverse events of tacrolimus were pruritus, skin burning (e.g., burning or warmth sensation, stinging), and skin infection (which included all cutaneous infections not otherwise specified, e.g., bacterial infections, molluscum, and pyoderma) [19]. So we changed the positive control to prednisolone in the further study (see Figure 1S in Supplementary Material available online at doi:10.1155/2012/743075).

To investigate the anti-inflammatory effect of AR, we estimated NO production in LPS-treated RAW 264.7 cells. It has been shown that physiological levels of NO can play an important role as an immune regulator, neurotransmitter, and a vasodilator in a variety of tissues. The high levels of NO produced by iNOS, however, have been defined as cytotoxic in inflammation and endotoxemia models [20]. Kang et al. reported that ethyl acetate fraction from AR had the anti-inflammatory effect [21]. We also confirmed that AR can inhibit NO production in LPS-treated RAW 264.7 cells in a dose-dependent manner.

The pathogenesis of AD is mediated by CD4^+^ T lymphocytes that produce Th2 cytokines. TARC and MDC function as selective chemoattractants that assist in the recruitment and migration of Th2 cells [22]. TARC and MDC are thought to play important roles in the development of skin diseases such as AD. In this study, we examined the effect of AR extract on hTARC production in TNF-*α*/IFN-*γ*-treated HaCaT cells and on the mRNA expression of TARC and MDC. We used forskolin and silymarin as positive controls to suppress inflammation. In another study, forskolin, a cAMP inhibitor, suppressed TARC and MDC levels in TNF-*α*/IFN-*γ*-treated HaCaT cells [23]. Silymarin was also shown to inhibit the AD-like skin lesions in *D. pteronyssinus*-induced NC/Nga mice and to suppress IL-4 and IgE levels in plasma [24]. In our study, the AR extract suppressed hTARC production in TNF-*α*/IFN-*γ*-treated HaCaT cells in a dose-dependent manner. AR and immunosuppressive controls were also able to inhibit TARC and MDC mRNA levels. We thought that MDC is less marginal effect than TARC level. AR may therefore regulate the recruitment of Th2-type cells to AD lesions by suppressing inflammatory mRNA expression. Some known antiallergic drugs are related to NF-*κ*B, STAT1, and p38 activation in activated macrophages and keratinocytes [25]. It will be necessary to assess NF-*κ*B and p38 in the cells used in our study to determine whether the activity of AR is associated with an anti-inflammatory or antiallergy effect. AR may, therefore, be an important new anti-inflammatory drug for the treatment of Th2-induced inflammation.

In conclusion, topical AR was shown to be effective in treating AD-like skin lesions in NC/Nga mice and inhibited AD-related gene (TARC and MDC) expression in TNF-*α*/IFN-*γ*-treated HaCaT cells. Topical application of AR may therefore be a novel approach to the treatment of AD.

## Supplementary Material

Efficacy of Angelicae Dahuricae Radix in NC/Nga mice.

## References

[1] Zheng X, Zhang X, Sheng X (2010). Simultaneous characterization and quantitation of 11 coumarins in Radix Angelicae Dahuricae by high performance liquid chromatography with electrospray tandem mass spectrometry. *Journal of Pharmaceutical and Biomedical Analysis*.

[2] Kang OH, Chae HS, Oh YC (2008). Anti-nociceptive and anti-inflammatory effects of angelicae dahuricae radix through inhibition of the expression of inducible nitric oxide synthase and NO production. *The American Journal of Chinese Medicine*.

[3] Li H, Dai Y, Zhang H, Xie C (1991). Pharmacological studies on the Chinese drug radix Angelicae dahuricae. *Zhongguo Zhongyao Zazhi*.

[4] Yi S, Cho JY, Lim KS (2009). Effects of angelicae tenuissima radix, angelicae dahuricae radix and scutellariae radix extracts on cytochrome P450 activities in healthy volunteers. *Basic and Clinical Pharmacology and Toxicology*.

[5] You L, An R, Wang X, Li Y (2010). Discovery of novel osthole derivatives as potential anti-breast cancer treatment. *Bioorganic and Medicinal Chemistry Letters*.

[6] Nie H, Shen YJ (2002). Effect of essential oil of radix angelicae dahuricae on *β*-endorphin, ACTH, NO and proopiomelanocortin of pain model rats. *Zhongguo Zhongyao Zazhi*.

[7] Kimura Y, Okuda H, Baba K (1997). Histamine-release effectors from Angelica dahurica var. dahurica root. *Journal of Natural Products*.

[8] Piao XL, Park IH, Baek SH, Kim HY, Park MK, Park JH (2004). Antioxidative activity of furanocoumarins isolated from Angelicae dahuricae. *Journal of Ethnopharmacology*.

[9] Akdis CA, Blaser K, Akdis M (2004). Apoptosis in tissue inflammation and allergic disease. *Current Opinion in Immunology*.

[10] Koo J, Arain S (1999). Traditional Chinese medicine in dermatology. *Clinics in Dermatology*.

[11] Jahnz-Rozyk K, Targowski T, Paluchowska E, Owczarek W, Kucharczyk A (2005). Serum thymus and activation-regulated chemokine, macrophage-derived chemokine and eotaxin as markers of severity of atopic dermatitis. *Allergy*.

[12] Kakinuma T, Nakamura K, Wakugawa M (2002). Serum macrophage-derived chemokine (MDC) levels are closely related with the disease activity of atopic dermatitis. *Clinical and Experimental Immunology*.

[13] Shimada Y, Takehara K, Sato S (2004). Both Th2 and Th1 chemokines (TARC/CCL17, MDC/CCL22, and Mig/CXCL9) are elevated in sera from patients with atopic dermatitis. *Journal of Dermatological Science*.

[14] Hashimoto S, Nakamura K, Oyama N (2006). Macrophage-derived chemokine (MDC)/CCL22 produced by monocyte derived dendritic cells reflects the disease activity in patients with atopic dermatitis. *Journal of Dermatological Science*.

[15] Lee M-Y, Seo C-S, Lee J-A (2011). Anti-asthmatic effects of Angelica dahurica against ovalbumin-induced airway inflammation via upregulation of heme oxygenase-1. *Food and Chemical Toxicology*.

[16] Hiroshi M, Naohiro W, Gregory P (1997). Development of atopic dermatitis-like skin lesion with IgE hyperproduction in NC/Nga mice. *International Immunology*.

[17] Minty A, Chalon P, Derocq JM (1993). Interleukin-13 is a new human lymphokine regulating inflammatory and immune responses. *Nature*.

[18] Sandoval-López G, Teran LM (2001). TARC: novel mediator of allergic inflammation. *Clinical and Experimental Allergy*.

[19] Hanifin JM, Paller AS, Eichenfield L (2005). Efficacy and safety of tacrolimus ointment treatment for up to 4 years in patients with atopic dermatitis. *Journal of the American Academy of Dermatology*.

[20] Moncada S, Palmer RMJ, Higgs EA (1991). Nitric oxide: physiology, pathophysiology, and pharmacology. *Pharmacological Reviews*.

[21] Kang OH, Lee GH, Choi HJ (2007). Ethyl acetate extract from Angelica Dahuricae Radix inhibits lipopolysaccharide-induced production of nitric oxide, prostaglandin E2 and tumor necrosis factor-*α* via mitogen-activated protein kinases and nuclear factor-*κ*B in macrophages. *Pharmacological Research*.

[22] Takashi N, Kunio H, Daisuke N (2004). Selective induction of Th2-attracting chemokines CCL17 and CCL22 in human B cells by latent membrane protein 1 of epstein-barr virus. *Journal of Virology*.

[23] Qi XF, Kim DH, Yoon YS (2009). The adenylyl cyclase-cAMP system suppresses TARC/CCL17 and MDC/CCL22 production through p38 MAPK and NF-*κ*B in HaCaT keratinocytes. *Molecular Immunology*.

[24] Kang JS, Yoon WK, Han MH (2008). Inhibition of atopic dermatitis by topical application of silymarin in NC/Nga mice. *International Immunopharmacology*.

[25] Hämäläinen M, Nieminen R, Vuorela P, Heinonen M, Moilanen E (2007). Anti-inflammatory effects of flavonoids: genistein, kaempferol, quercetin, and daidzein inhibit STAT-1 and NF-*κ*B activations, whereas flavone, isorhamnetin, naringenin, and pelargonidin inhibit only NF-*κ*B activation along with their inhibitory effect on iNOS expression and NO production in activated macrophages. *Mediators of Inflammation*.

