# Supplementary material for: Angelicae Dahuricae Radix Inhibits Dust Mite Extract-Induced Atopic Dermatitis-Like Skin Lesions in NC/Nga Mice
Source: Evid Based Complement Alternat Med. 2012 Feb 12;2012:743075. doi: 10.1155/2012/743075 (PMC3290843; doi:10.1155/2012/743075)
Supplement: Supplementary file 1 — Efficacy of Angelicae Dahuricae Radix in NC/Nga mice. [file 743075.f1.docx]

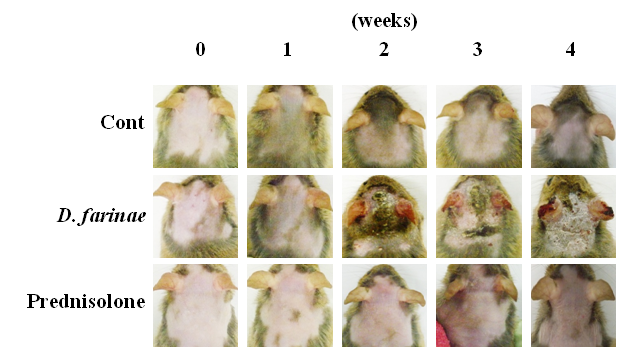


F_IGURE_ 1S: Histological changes of treated backs and ears in NC/Nga mice. Representative features and histological changes following consecutive administration of prednisolone to *Dermatophagoides farinae*-induced AD-like lesions on the back and ears. The images show the back and ears 4 weeks after sensitization.
